# Spectral affinity in protein networks

**DOI:** 10.1186/1752-0509-3-112

**Published:** 2009-11-29

**Authors:** Konstantin Voevodski, Shang-Hua Teng, Yu Xia

**Affiliations:** 1 Department of Computer Science, Boston University, Boston, MA 02215, USA; 2 Microsoft Research New England, Cambridge, MA 02142, USA; 3 Bioinformatics Graduate Program, Boston University, Boston, MA 02215, USA; 4 Department of Chemistry, Boston University, Boston, MA 02215, USA

## Abstract

**Background:**

Protein-protein interaction (PPI) networks enable us to better understand the functional organization of the proteome. We can learn a lot about a particular protein by querying its neighborhood in a PPI network to find proteins with similar function. A spectral approach that considers random walks between nodes of interest is particularly useful in evaluating closeness in PPI networks. Spectral measures of closeness are more robust to noise in the data and are more precise than simpler methods based on edge density and shortest path length.

**Results:**

We develop a novel affinity measure for pairs of proteins in PPI networks, which uses personalized PageRank, a random walk based method used in context-sensitive search on the Web. Our measure of closeness, which we call *PageRank Affinity*, is proportional to the number of times the smaller-degree protein is visited in a random walk that restarts at the larger-degree protein. PageRank considers paths of all lengths in a network, therefore *PageRank Affinity *is a precise measure that is robust to noise in the data. *PageRank Affinity *is also provably related to cluster co-membership, making it a meaningful measure. In our experiments on protein networks we find that our measure is better at predicting co-complex membership and finding functionally related proteins than other commonly used measures of closeness. Moreover, our experiments indicate that *PageRank Affinity *is very resilient to noise in the network. In addition, based on our method we build a tool that quickly finds nodes closest to a queried protein in any protein network, and easily scales to much larger biological networks.

**Conclusion:**

We define a meaningful way to assess the closeness of two proteins in a PPI network, and show that our closeness measure is more biologically significant than other commonly used methods. We also develop a tool, accessible at http://xialab.bu.edu/resources/pnns, that allows the user to quickly find nodes closest to a queried vertex in any protein network available from BioGRID or specified by the user.

## Background

Networks are often used to represent a system where the nodes are a set of agents, and the edges are the relationships/interactions between those agents. We can then use the network topology to find out more about the nodes and the relationships between them. For example, we can find vertices central to the network, which is useful for biological [1,2] and social networks [3]. In addition, we can use the network topology to find communities, in the context of the Internet [4-6], and social and biological networks [7-9]. It is also useful to consider relationships between pairs of vertices. For an interacting pair, we can look at how essential this interaction is to the network [10], and use this information for community detection [11,12], or to find properties of these pairs [13].

Moreover, we can use the graph topology to evaluate the *closeness *of pairs of vertices, which can provide additional insight into the structure of the network. In many networks most node pairs are not connected, but it is still meaningful to consider how nodes that are not directly connected relate to each other. A lack of an interaction may be due to physical constraints on the nodes, such as an airport only being able to support service to so many other airports, or a person only being capable of knowing so many other people. However, it may also simply be due to noise in the network, such as the extremely high false negative rate in protein networks [14,15]. Conversely, even among node pairs that are connected it is still meaningful to see which pairs are closer to each other. For example, we may consider a pair of vertices in the same cluster in the graph to be closer than a pair that is not. And once again, some connections may simply be due to noise.

The simplest notion of distance between two nodes in a network is the length of the shortest path between them. However, this is an imprecise measure, especially for small-world graphs where the longest path between any two nodes is very short [16,17]. As an extreme example, consider large social networks, where it is speculated that the longest distance between any two people is six [18]. Shortest path distances consider a single path in assessing the closeness of two nodes. If we use a measure that takes all the paths in the network into account, we should be able to compute distances that are more meaningful.

Here we consider the problem of assessing the closeness of two proteins in a protein-protein interaction (PPI) network. There has been considerable work done in this area, where measures of interconnectedness between protein pairs have been used to find functionally similar proteins [19-22]. Different notions of interconnectedness have also been used to predict false negative interactions in protein networks [23]. All of these measures consider the density of interactions in the immediate neighborhood of two proteins, and some also normalize by the number of interactions of each protein, or the number of interactions in the neighborhood expected by chance.

A problem that is related to computing the distance between two proteins is finding the closest neighbors of a set of proteins. This is addressed in [24] by generalizing pairwise notions of interconnectedness. A similar problem is considered in the context of probabilistic PPI networks, where reachability [25] and shortest path distance [26] (in instantiated networks) are used to recover protein complexes when only some of their proteins are known. Discovering protein complexes from protein network topology is itself a very well-studied problem, and is usually addressed by clustering [27-31].

## Contribution

In this work we develop a novel method to evaluate the closeness of proteins in a PPI network. We also create a tool that allows the user to quickly find nodes closest to a queried protein in any PPI network available from BioGRID or specified by the user. Our measure of closeness uses personalized PageRank, which was introduced by [32], and has been used for context-sensitive search on the Web [33,34]. We define the *PageRank Affinity *of two proteins *a *and *b *to be the minimum of pr(*a *→ *b*) and pr(*b *→ *a*), where pr(*a *→ *b*) is the amount of PageRank that *a *contributes to *b *in the PPI network, which is proportional to the number of times *b *is visited in a random walk on the network that restarts at *a*. We show that our closeness measure is more biologically meaningful than other commonly used methods in terms of predicting co-complex membership and correlation with functional distance. Moreover, we show that our method is very resilient to noise in the data. We also provide intuition for why *PageRank Affinity *is meaningful in protein networks by stating some properties of PageRank vectors.

## Methods

### Preliminaries

We model a protein interaction network as an undirected, unweighted graph where the nodes are the proteins, and two nodes are connected by an edge if the corresponding proteins are annotated as interacting with each other.

Formally, a graph is given by a set of vertices *V *and a set of edges *E*. The degree of a node *u *∈ *V*, denoted by *d*(*u*), is the number of edges adjacent to *u*. A graph is often represented by its adjacency matrix. The adjacency matrix of a graph *G *= (*V, E*) is defined by

We can learn a lot about the structure of a graph by taking a random walk on it. A random walk is a process where at each step we move from some node to one of its neighbors. The transition probabilities are given by edge weights, so in the case of an unweighted network the probability of transitioning from *u *to any adjacent node is 1/*d*(*u*). Thus the transition probability matrix (often called the random walk matrix) is the normalized adjacency matrix where each row sums to one:

Here the *D *matrix is the degree matrix, which is a diagonal matrix given by

In a random walk it is useful to consider a probability distribution vector *p *over all the nodes in the graph. Here *p *is a row vector, where *p*(*u*) is the probability that we are at node *u *and Σ_*u*∈*V *_*p*(*u*) = 1. Because we transition between nodes with probabilities given by *W*, if *p*_*t *_is the probability distribution vector at time *t*, then *p*_*t*+1 _= *p*_*t*_*W*.

### PageRank

A PageRank vector pr_*α *_(*s*) is the steady state probability distribution of a random walk with restart probability *α*. The starting vector *s *gives the probability distribution for where the walk transitions after restarting. Formally, pr_*α *_(*s*) is the unique solution of the linear system

The PageRank vector with a uniform vector for *s *gives the global PageRank of each vertex. PageRank with non-uniform starting vectors is known as personalized PageRank.

Here we always use a starting vector that has all of its probability in one vertex, defined as follows:

pr_*α *_(*e*_*u*_) is thus the steady-state probability distribution of a walk that always returns to *u *at restart, and we will refer to it as the personalized PageRank vector of *u*. We will use pr_*α *_(*e*_*u*_) [*v*] to denote the amount of probability that *v *has in pr_*α *_(*e*_*u*_), and use a shorthand of pr(*u *→ *v*) for this quantity, dropping the *α *in the subscript because in our computations it is always fixed. As pointed out in [35], *v*'s global PageRank, denoted by PR(*v*), satisfies

Thus pr(*u *→ *v*) can be thought of as the contribution that *u *makes to the PageRank of *v*.

### PageRank Affinity

For two vertices *u *and *v *we define their *PageRank Affinity *to be the minimum of the PageRank that *u *contributes to *v *and *v *contributes to *u*:

This quantity can be computed by solving the PageRank equation for pr_*α *_(*e*_*u*_) and pr_*α *_(*e*_*v*_), and reporting the minimum of the two PageRank contributions. The restart probability of the random walk (*α*) must be greater than 0 to ensure that pr_*α *_(*e*_*u*_) and pr_*α *_(*e*_*v*_) have unique solutions, and must be much smaller than 1 to prevent the random walk from returning too often to the starting vertex and being too local. We set *α *to 0.15, which is typical for computations of PageRank.

### Approximate PageRank Affinity

We can also use approximate PageRank to compute closeness between nodes. While it is possible to compute exact PageRank vectors for smaller graphs by solving the PageRank equation, it is computationally infeasible to do this for larger networks. To calculate approximate PageRank, we use the ApproximatePR algorithm from [36], which computes an ϵ-approximate PageRank vector for a random walk with restart probability *α *in time *O*(). An ϵ-approximate PageRank vector for pr_*α *_(*s*), denoted by _α_(*s*), satisfies(1)

for any subset of vertices *S*, where *p *[*S*] = Σ_*v*∈*S *_*p *[*v*], and vol(*S*) = Σ_*v*∈*S *_*d*(*v*). In other words, the amount of error in the approximate PageRank vector for any subset of vertices is at most the product of ϵ and the sum of degrees of its nodes.

#### Algorithm Description

We develop an algorithm that approximates *PageRank Affinity*, which uses ApproximatePR as a subroutine. Our *approximatePRaffinity *algorithm takes a queried vertex *v*, approximation parameter ϵ, and integer *k *as input, and returns the *k *nodes closest to *v *in the graph. The algorithm is outlined below.

**Algorithm 1 **approximatePRaffinity(*v*, ϵ, *k*)

   (*e*_*v*_) = ApproximatePR(*v*, ϵ)

   **for **each *u ***do**

      (*v *→ *u*) = (*e*_*v*_) [*u*]

   **end for**

   **for **each *u ***do**

      (*u *→ *v*) = (*v *→ *u*)

   **end for**

   **for **each *u ***do**

      **affinity**(*u*) = min((*u *→ *v*), (*v *→ *u*))

   **end for**

   return the *k *vertices with highest **affinity **scores

We first compute an approximate personalized PageRank vector of *v*, denoted by (*e*_*v*_), to approximate the amount of PageRank that *v *gives to each vertex *u*, denoted by (*v *→ *u*). We then use the observation that for undirected graphs(2)

to approximate the PageRank contribution of each vertex in the graph to *v*. We then calculate the *affinity *to *v *of each vertex *u *as

and return the *k *nodes with highest *affinity *values. Equation 2 follows from the discussion of computing PageRank contributions in the time-reverse Markov chain in [35], and the fact that in an undirected graph the amount of probability that a vertex has in the stationary distribution of a random walk is proportional to its degree.

It follows from Equation 1 that the amount of error in the probability that *u *has in the approximate personalized PageRank vector of *v *is at most ϵ·*d*(*u*):(3)

We denote by pr-aff(*u*, *v*) the exact *PageRank Affinity *of *u *and *v*, and by (*u*, *v*) the *Approximate PageRank Affinity *computed by *approximatePRaffinity*. Using Equations 2 and 3 we can verify that the amount of error in the *Approximate PageRank Affinity *of vertices *u *and *v *is at most the product of ϵ and the larger of their degrees:

#### Runtime Analysis

The approximate PageRank vector computed by ApproximatePR has few non-zero entries. This saves computation time because we do not need to consider vertices with 0 probability in the approximate PageRank vector (they have an *affinity *of 0). Let us call the support of probability distribution vector *p*, denoted by Supp(*p*), the set of all vertices that have non-zero probability in *p*:

ApproximatePR computes an approximate PageRank vector with small support, which is useful for large graphs that have many vertices. More specifically, the number of non-zero entries in the approximate PageRank vector is less than :

Thus the exact runtime of *approximatePRaffinity *is the time necessary to compute (*e*_*v*_), which takes *O*(), plus the time necessary to compute the *affinity *to *v *of each vertex in Supp((*e*_*v*_)), which is linear in the size of the support set, plus the time necessary to find the *k *vertices with largest *affinity *scores, which takes at most *k*·, giving a total runtime of *O*( + ). Moreover, if we treat *α *as a constant in this analysis (because we always set it to 0.15), this expression simplifies to *O*().

### Properties of PageRank Vectors

It is well-known that a PageRank vector can be expressed as a weighted average of random walk vectors [36]:(4)

The *sW*^*t *^term gives the probability distribution of the random walk after *t *steps. Equation 4 thus shows that in computing PageRank we consider paths of all lengths, with less weight given to longer paths based on the value of *α*.

Another important property of PageRank vectors is that if *u *and *v *are in the same cluster, both pr(*u *→ *v*) and pr(*v *→ *u*) are likely to be high. The quality of a cluster *C *is measured by proportion of outgoing edges, known as conductance, which we denote by Φ(*C*). A cluster of lower conductance is better because its nodes are more connected among themselves than they are with the other nodes in the graph. It is proved in [36] that for any set *C*, there is a subset of vertices *C' *⊆ *C*, such that for any vertex *u *∈ *C'*, the personalized PageRank vector of *u*, denoted by pr_*α*_(*e*_*u*_), satisfies

In other words, pr(*u *→ *v*) = pr_*α*_(*e*_*u*_) [*v*] is high on average if *u *and *v *are in the same good (low-conductance) cluster *C *and *u *∈ *C'*. Moreover, the set *C' *is large, as the sum of degrees of its nodes, denoted by vol(*C'*), satisfies vol(*C'*) ≥ vol(*C*)*/*2.

### Other Measures of Closeness

In our experiments on protein networks, we compare *PageRank Affinity *and *Approximate PageRank Affinity *with several other measures of closeness, which are described below. Some of these measures assign an affinity score to each pair of vertices, while others simply order pairs by their closeness.

#### Shortest Path and Shortest Path Multiplicity

The shortest path closeness of two vertices is the inverse of the length of the shortest path between them. However, using the length of the shortest path does not allow for much granularity, so we also consider the multiplicity of the shortest path to break ties between pairs that are the same distance apart.

#### Common Neighbors

A very intuitive measure of closeness of two vertices is the number of neighbors that they share in the graph. In our experiments, we notice that in addition to counting common neighbors, it also helps to take into account whether the two nodes are directly connected, by adding a small constant to their closeness score if this is the case.

#### Partitioning

We also compare with another measure of closeness, motivated by efforts to partition PPI networks and determine overlap with known protein complexes. It is observed that the densest clusters are often the ones that overlap most with known complexes [30]. Therefore, we partition the protein network, and score pairs of vertices that are in the same cluster by the edge density of the cluster. To partition the network, we use Metis [37], a widely used algorithm that finds high-quality, balanced clusters in the graph. Once we partition the network, we consider protein pairs in denser clusters closer than pairs in less dense clusters, because pairs in denser clusters are more likely to be part of the same functional unit. Of course, this approach only allows us to consider a small fraction of the pairs, because we have no way to evaluate the closeness of two proteins assigned to different clusters.

#### Cliques and *k*-cores

In addition to partitioning the graph and evaluating the edge density of each cluster, we can also search for dense components directly by enumerating maximum cliques and finding *k*-cores. A *k*-core is a vertex-induced subgraph where the degree of each node is at least *k *[38]. We then consider pairs that are part of a larger clique closer than pairs that are part of a smaller clique, and consider pairs that are part of an *m*-core closer than pairs that are part of an *n*-core if *m *>*n*. However, once again, these measures allow us to evaluate the closeness of only a small number of pairs in the network.

#### Commute time

Another way to assess the closeness of two nodes using a random walk on the graph is to consider the inverse of the commute time between them. The commute time between vertices *u *and *v *is the expected number of steps taken for a random walk from *u *to reach *v *and return, which is computed as described in [39].

## Results

We develop a tool, which is accessible at http://xialab.bu.edu/resources/pnns, that quickly finds nodes closest to a queried protein in any protein-protein interaction (PPI) network available from BioGRID. Our tool implements the *approximatePRaffinity *algorithm described in Methods, and returns a list of proteins sorted by their *Approximate PageRank Affinity *to the queried protein, along with the affinity scores (normalized and rounded to one significant figure). The user can specify a protein network by selecting an organism and a set of interaction types. In addition, one may upload a custom (undirected) network, which may be weighted. Our application is also available as a command-line executable.

**Figure 1:**
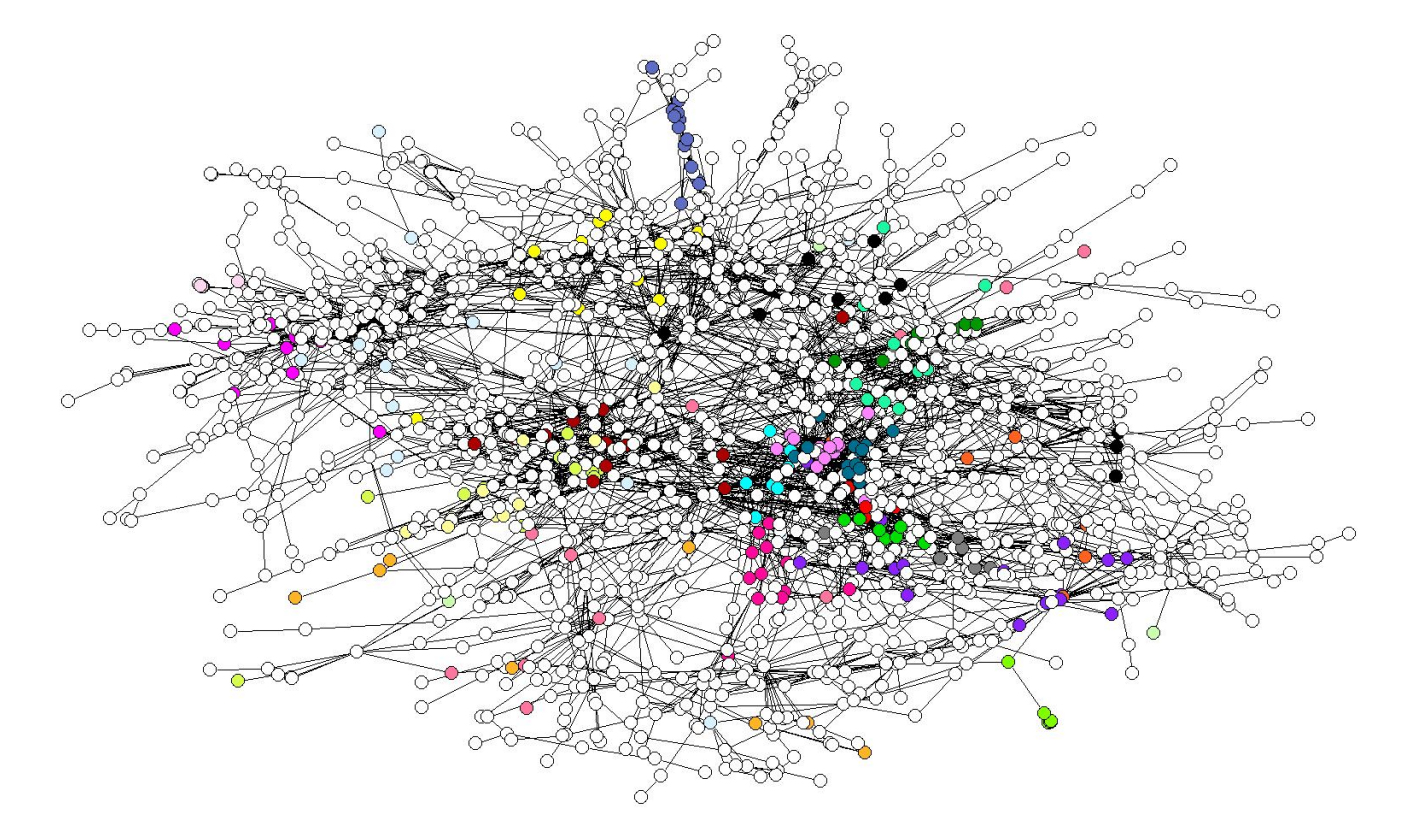
**A view of the protein complexes in one of the PPI networks**. The AC-Western network, with proteins in the same complex labeled with the same color.

To assess the meaning of our closeness measure in protein networks we use protein complex annotation from [40], and functional distance data from [41]. We consider these datasets "gold standard" measures of protein functional similarity because they are based on information that is manually curated. A good measure of closeness in a protein-protein interaction network should be consistent with these data: we expect many of the closest nodes in the network to be in the same protein complex, and more generally have lower functional distances. Figure 1 displays one of the protein networks used in our study, with proteins annotated to be in the same complex labeled using the same color: we can see that a lot of the clusters in the network indeed contain proteins that belong to the same complex.

We evaluate the closeness of protein pairs in PPI networks from BioGRID [42], Version 2.0.44. BioGRID is a repository that lists interacting protein pairs, and for each pair gives the experimental method used to detect the interaction. We use three different networks in our study, which differ in the type of experiment used to detect the interactions. One of the networks is formed from interactions detected by Affinity Capture-Western experiments (which we refer to as **AC-Western**), and the other two are from Affinity Capture-MS (referred to as **AC-MS**), and **Two-Hybrid **experiments. In each network, we use *PageRank Affinity *and other measures described in Methods to rank protein pairs by closeness, in order to determine which measures are more biologically significant.

To calculate the *PageRank Affinity *of all pairs of nodes in a network, we compute a personalized PageRank vector of each vertex, and then calculate a *PageRank Affinity *score for each pair from their personalized PageRank vectors, as described in Methods. In order to see if we get similar results with the quicker approximation method that our tool implements, in each network we also calculate the *Approximate PageRank Affinity *of protein pairs by running the *approximatePRaffinity *algorithm from each vertex.

### Predicting Co-Complex Membership

**Figure 2:**
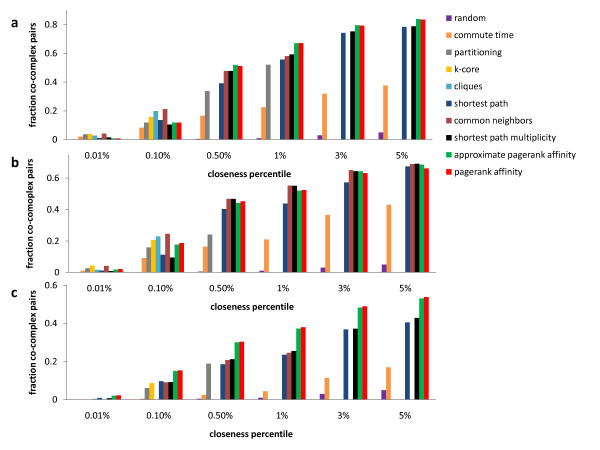
**Which measure of closeness is best at predicting co-complex membership? **The number of co-complex pairs (as a fraction of the total number of co-complex pairs in the network) among pairs in the top percentile of each closeness ranking is displayed. Higher values indicate measures that are more biologically meaningful. (a) The results for the AC-Western network. (b) The results for the AC-MS network. (c) The results for the Two-Hybrid network.

We first investigate which measure of closeness is best at predicting co-complex membership. In every network, we compare how many co-complex pairs are in the top percentile of each closeness ranking. We also calculate the number of co-complex pairs that we expect in each percentile by chance, to see how statistically significant the results are. Figure 2 displays the results of our computational experiment. There are three panels: one for each PPI network that we study. Bars of different color are used to represent the results for the different closeness measures compared. In each sub-figure the x-axis lists different percentiles of the closeness rankings, and the y-axis lists the fraction of the total number of co-complex pairs in the network contained in the top percentile of a particular ranking. For example, there are 2851 co-complex pairs among the proteins in the AC-Western network. When we examine the top 1% pairs with highest *PageRank Affinity *in this PPI network, there are 1915 co-complex pairs among them, which constitutes a  = 0.67 fraction displayed in the figure.

Figure 2c shows that in the Two-Hybrid network there are more co-complex pairs among protein pairs with high *PageRank Affinity*. The same is true for the AC-Western network, although the contrast with other measures of closeness is smaller (Figure 2a). The picture is different for the AC-MS network, as common neighbors and shortest path multiplicity are as effective as *PageRank Affinity *in predicting co-complex pairs (Figure 2b). We also note that in all three networks we do not lose much by approximating *PageRank Affinity *rather than computing it exactly.

From Figure 2 we also see that few co-complex pairs are considered close using partitioning. Partitioning seeks out clusters that are balanced in size, which often hurts their quality. *PageRank Affinity *is also related to cluster co-membership (see Methods), but considers whether two nodes are part of a local cluster rather than a cluster in a global partition of the graph. The closeness of two nodes is then proportional to the quality of this local cluster, which does not depend on a partition of the entire network, which is less relevant. Therefore *PageRank Affinity *is a more flexible measure that better reflects whether two nodes are part of some quality cluster in the network.

### Correlation with Functional Distance

In addition to using protein complex annotation, we also use functional distance data to see which measures of closeness are more biologically relevant. To calculate functional distances, we use a measure based on GO (Gene Ontology) biological process annotation. The method of [41] provides a very sensitive measure of functional distance because it considers all known functions of a pair of proteins in assigning them a score.

We first investigate whether there is any global correlation between the functional distances and any measure of closeness. In each network we rank all protein pairs by functional distance, and compute correlation with the closeness ranking of each measure using the Pearson Correlation Coefficient [43]. However, we find that there is very little global correlation between functional distances and any measure of closeness. This is not surprising because functional annotation and protein-protein interaction data are fundamentally very different.

**Figure 3:**
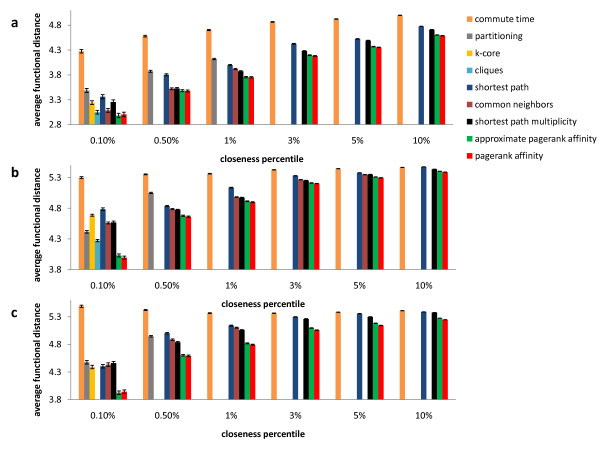
**Which measure of closeness best correlates with functional distance? **The average functional distance of pairs in the top percentile of each closeness ranking is displayed. Lower values indicate measures that are more biologically meaningful. (a) The results for the AC-Western network. (b) The results for the AC-MS network. (c) The results for the Two-Hybrid network. The average functional distance of two proteins in the genome is 5.8.

Therefore we perform a different analysis, where we simply average the functional distances of the protein pairs in the top *k *percent of each closeness ranking, for different values of *k*. In our calculations of functional distance we take the logarithm (base 10) of the functional distance score from [41]; a lower value indicates a closer functional relationship. We notice that for most closeness rankings the average functional distance is worse for protein pairs that are further away, as expected. The results of our experiment are presented in Figure 3. Once again, there are three panels, one for each protein network that we study, and bars of different color are used to represent the results for the different closeness measures compared. In each sub-figure the x-axis lists different percentiles of the closeness rankings, and the y-axis displays the average functional distance of protein pairs in the top percentile of a particular ranking.

Figure 3 shows that in all three networks protein pairs with high *PageRank Affinity *are more functionally related (have smaller functional distances). Once again, *Approximate PageRank Affinity *is almost as biologically meaningful, significantly outperforming other measures. We note that *Approximate PageRank Affinity *outperforms closeness based on large clique membership, which is intuitively a very good measure of closeness. Moreover, it is not possible to find large cliques in real time, whereas *Approximate PageRank Affinity *takes seconds to compute and will scale to larger protein networks as the size of the protein interaction data continues to grow.

### Robustness to Noise

A closeness measure is particularly useful if it tells us something about the true structure of the network in the presence of noise. The interactions in PPI networks are not random: proteins function in modules, therefore we should be able to use the community structure of the network to identify true positive and true negative interactions, unless this structure is completely destroyed by noise.

As described in Methods, our measure of closeness is provably related to cluster co-membership, therefore we expect it to be resilient to noise if the network has strong community structure. To test this hypothesis, we conduct an experiment where we build a PPI network from the complex annotation in [40], add noise to it by adding/removing edges, and then evaluate the closeness of true positive and true negative interactions in the noisy network using *PageRank Affinity*. To build the protein network we connect two proteins by an edge if they are annotated as co-complexed in [40]. We only consider protein complexes of size ≥30, which results in a network with 250 nodes and 6913 edges.

To evaluate the resilience of a closeness measure to noise, we use a metric that considers the closeness of nodes in the noisy network, and counts how often true positive interactions are closer than true negative interactions. More specifically, let *G *= (*V, E*) be the true network and  = (*V*, ) be the modified (noisy) network. We add noise to the network by randomly choosing *r*_1 _* |*E*| of the true positive interactions and removing them, and randomly choosing *r*_2 _* |*E*| of the true negative interactions and adding them. We then consider node pairs *p*_1 _= (*u*_1_, *v*_1_) ∈ *V *× *V *and *p*_2 _= (*u*_2_, *v*_2_) ∈ *V *× *V*, such that one of them is connected in the true network and the other is not: either (*u*_1_, *v*_1_) ∈ *E *and (*u*_2_, *v*_2_) ∉ *E *or (*u*_1_, *v*_1_) ∉ *E *and (*u*_2_, *v*_2_) ∈ *E*. We call (*p*_1_, *p*_2_) concordant if the connected pair is closer in the noisy network than the disconnected pair, and discordant otherwise.

For a particular experiment, where we take the true network, add noise to it, and calculate the closeness of node pairs in the modified network, we use *n*_*c *_to denote the number of concordant pairs and *n*_*d *_to denote the number of discordant pairs. We then calculate a **robustness score **for a similarity measure as  ∈ [-1, 1]. A score of 1 indicates that each true positive interaction is closer in the noisy network than each true negative interaction, and a score of -1 indicates that each true positive interaction is further in the noisy network than each true negative interaction.

**Figure 4:**
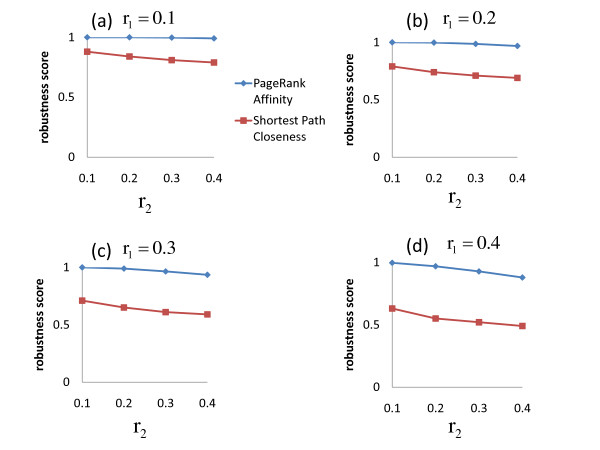
**How robust is PageRank Affinity to noise? **We add noise to a network represented by a graph *G *= (*V, E*) by randomly choosing *r*_1 _* |*E*| of the true positive interactions and removing them, and randomly choosing *r*_2 _* |*E*| of the true negative interactions and adding them. We calculate the **robustness score **of *PageRank Affinity *by computing the *PageRank Affinity *of each pair of vertices in the noisy network, and counting how often true positive interactions score higher than true negative interactions. As a base for comparison, we also compute the robustness score of shortest path closeness (with ties in distance broken randomly). (a) The results for *r*_1 _= 0.1 and varying values of *r*_2_. (b) The results for *r*_1 _= 0.2 and varying values of *r*_2_. (c) The results for *r*_1 _= 0.3 and varying values of *r*_2_. (d) The results for *r*_1 _= 0.4 and varying values of *r*_2_.

Figure 4 displays the results of our computational experiment. We vary *r*_1 _and *r*_2 _to create several noisy networks, and record the robustness score of *PageRank Affinity *for different levels of noise. As a base for comparison, we also compute the robustness score of shortest path closeness, where all ties in distance are broken randomly. From Figure 4 we notice that when *r*_1 _= *r*_2 _= 10%, *PageRank Affinity *scores a perfect 1, indicating that even though we have added considerable noise to the network, we can still perfectly distinguish between true positive and true negative interactions: if we know the number of true edges, we can simply take the highest scoring |*E*| node pairs in the noisy network to recover the true interactions. Even as we increase the amount of noise considerably, the robustness score for *PageRank Affinity *remains very high, while for shortest path closeness it is much lower.

This experiment provides insight into why our closeness measure is effective at identifying co-complex protein pairs in PPI networks. The protein networks used in our study are similar to , the noisy network in our experiment: they are formed from a true set of protein-protein interactions given by the protein complexes, which is altered by noise due to the nature of the experiments used to detect the interactions. Still, the noisy network preserves some of the community structure, and many of the protein pairs that have high *PageRank Affinity *are true interactions, which are the co-complex pairs.

With this in mind, we further investigate whether our measure can be used to detect false negative interactions in PPI data by examining non-interacting pairs with highest *PageRank Affinity *in each protein network used in our study. For each pair, we look for other evidence of interaction between the two proteins using complex annotation from [40], and also examine whether the two proteins are annotated as interacting in BioGRID using other experimental methods.

We find that for many of the disconnected (not directly connected) pairs with highest *PageRank Affinity *there are other (often multiple) sources of evidence of interaction between the two proteins. The results are presented in Additional files 1, 2, 3, where we consider the 20 disconnected protein pairs with highest *PageRank Affinity *in each network. Additional file 1 shows that when we examine the 20 disconnected pairs with highest *PageRank Affinity *in the AC-Western network, we find evidence that 14 of them are in fact interacting. For the ACMS and Two-Hybrid networks we find 5 and 7 such pairs (out of 20), respectively (see Additional files 2 and 3). These numbers are all much higher than expected by chance (for each network the probability that a non-interacting pair chosen at random is annotated as an interaction elsewhere is less than 0.01).

## Discussion

### Defining PageRank Affinity

To determine the closeness of proteins *a *and *b *in a PPI network, we compute two PageRank contributions *c*_1 _= pr(*a *→ *b*) and *c*_2 _= pr(*b *→ *a*) (see Methods). To better understand how to combine the two contributions into a single score, we consider different possibilities, and see which ones are more biologically relevant in terms of predicting co-complex membership and correlation with functional distance (using the same experiments described in the Results). We first try combinations of the form

for different values of *β*. Here *β *= 0 corresponds to taking the minimum of *c*_1 _and *c*_2_, *β *= 1/2 corresponds to taking the arithmetic mean, and *β *= 1 corresponds to taking the maximum of the two contributions. In addition, we try combinations of the form of

for different values of *β*. Here *β *= 0 corresponds to taking the minimum of the two contributions, *β *= 1/2 corresponds to taking the geometric mean, and *β *= 1 to taking the maximum.

When we evaluate different ways of combining the two PageRank contributions we notice that taking the minimum of *c*_1 _and *c*_2 _better predicts co-complex membership in two out of the three networks. Moreover, using the minimum of the two contributions gives a measure that is more correlated with functional distance in all three networks.

The complete results of our experiments are presented in Additional files 4 and 5. Additional file 4 displays the results of evaluating different ways of combining the two PageRank contributions using a weighted arithmetic mean, while Additional file 5 displays the results of evaluating different ways of combining the two values using a weighted geometric mean. There are 6 panels in each figure: the first three display a comparison in terms of predicting co-complex membership (one sub-figure for each protein network that we study), and the next three display a comparison in terms of correlation with functional distance (one sub-figure for each protein network). For the first three panels higher values are better because they indicate more co-complex pairs that are close, while for the next three panels lower values are better because they indicate that pairs that are close have low functional distances, and are thus more functionally related.

Our experiments indicate that taking the minimum of pr(*a *→ *b*) and pr(*b *→ *a*) gives a more biologically meaningful measure of closeness. Furthermore, we know that in an undirected network pr(*a *→ *b*) = pr(*b *→ *a*), where *d*(*a*) denotes the degree of *a*. Therefore using the minimum of the two contributions is equivalent to reporting pr(*a *→ *b*) if *d*(*a*) ≥ *d*(*b*), and pr(*b *→ *a*) otherwise. Thus our measure of closeness considers a random walk on the protein network from the larger-degree protein to the smaller-degree protein, which is a sound approach because proteins with large degree are easily reachable from most other proteins in a random walk regardless of their identity.

### Advantages of PageRank Affinity

As discussed in Methods, node pairs that are part of the same cluster in a graph will likely have higher *PageRank Affinity*. This property makes it a very relevant measure for protein networks because we expect pairs of proteins in the same cluster to be more functionally related, and be more likely to be in the same protein complex. Therefore it is not surprising that *PageRank Affinity *outperforms other measures of closeness in terms of correlation with functional distance and predicting co-complex membership.

In addition, *PageRank Affinity *is a very precise measure of closeness, and is robust to noise in the data. As discussed in Methods, personalized PageRank takes into account all paths between two nodes in a graph (with more weight given to shorter paths), by considering an arbitrarily long random walk from the starting vertex. This allows for more precision in assessing closeness, which is especially useful in protein networks, where most protein pairs are very close to each other in terms of shortest path distance. Moreover, considering all paths between two vertices makes *PageRank Affinity *very robust to noise in the data, because a few extraneous or missing edges are less likely to make a big difference in evaluating the closeness of two nodes. We also have experimental evidence to support this claim, as *PageRank Affinity *performs very well in distinguishing between true positive and true negative interactions in our simulated noisy networks.

### A Tool to Compute PageRank Affinity

One of our goals is to use our closeness measure to develop a practical tool that can be used to quickly find proteins closest to a queried vertex in a PPI network. We can compute the *PageRank Affinity *of one vertex to all other vertices in the network by solving a single PageRank equation (see Methods). While it is possible to do this in real time for most protein networks formed from interactions listed in BioGRID today, as the size of the data continues to grow this computation will become more challenging. Moreover, even right now some of the protein networks from BioGRID (constructed from the union of several interaction types) are already too big to compute *PageRank Affinity *exactly in real time.

Our approximation algorithm, on the other hand, allows us to easily manage the tradeoff between computation time and the quality of the produced output. In our experiments we have shown that when we approximate *PageRank Affinity*, the quality of the output does not decrease much. Our tool works quickly on datasets currently available in BioGRID, and easily scales to much larger protein networks and other biological networks.

## Conclusion

We develop a method to evaluate the closeness of two proteins in a PPI network, and show that it is biologically meaningful in terms of predicting co-complex membership and correlation with functional distance. Moreover, we create a tool that can be used to quickly find nodes closest to a queried vertex in any protein network available from BioGRID or specified by the user.

## Authors' contributions

KV, SHT, and YX conceived and designed the experiments. KV performed the experiments and analyzed the data. KV, SHT, and YX drafted and edited the manuscript. All authors read and approved the final manuscript.

## Supplementary Material

Additional file 1 **Predicted false negative interactions in the AC-Western network**. The table lists disconnected node pairs with highest *PageRank Affinity *in the AC-Western network, along with evidence for the existence of each interaction. Each row specifies a pair of proteins and the rank of their *PageRank Affinity *score (**closeness rank**). For each pair we list evidence of the existence of this interaction in the **evidence of interaction** column by writing **co-complexed** if the two proteins are annotated as co-complexed in [40], and writing **ac-western**, **ac-ms**, **two-hybrid**, and **other** if the pair is listed as interacting in BioGRID using Affinity Capture-Western, Affinity Capture-MS, Two-Hybrid, or any other type of experiment. Click here for file

Additional file 2 **Predicted false negative interactions in the AC-MS network**. The table lists disconnected node pairs with highest *PageRank Affinity *in the AC-MS network, along with evidence for the existence of each interaction. Each row specifies a pair of proteins and the rank of their *PageRank Affinity *score (**closeness rank**). For each pair we list evidence of the existence of this interaction in the **evidence of interaction **column by writing **co-complexed **if the two proteins are annotated as co-complexed in [40], and writing **ac-western**, **ac-ms**, **two-hybrid**, and **other **if the pair is listed as interacting in BioGRID using Affinity Capture-Western, Affinity Capture-MS, Two-Hybrid, or any other type of experiment. Click here for file

Additional file 3 **Predicted false negative interactions in the Two-Hybrid network**. The table lists disconnected node pairs with highest *PageRank Affinity *in the Two-Hybrid network, along with evidence for the existence of each interaction. Each row specifies a pair of proteins and the rank of their *PageRank Affinity *score (**closeness rank**). For each pair we list evidence of the existence of this interaction in the **evidence of interaction** column by writing **co-complexed **if the two proteins are annotated as co-complexed in [40], and writing **ac-western**, **ac-ms**, **two-hybrid**, and **other **if the pair is listed as interacting in BioGRID using Affinity Capture-Western, Affinity Capture-MS, Two-Hybrid, or any other type of experiment. Click here for file

Additional file 4 **Evaluating different ways of defining *PageRank Affinity *by using a weighted arithmetic mean to combine the two PageRank contributions**. We evaluate different ways of combining *c*_1 _= pr(*a *→ *b*) and *c*_2 _= pr(*b *→ *a*) using a weighted arithmetic mean: *β*·max(*c*_1_, *c*_2_) + (1 - *β*)·min(*c*_1_, *c*_2_). Each value of *β *gives a distinct closeness measure (represented by bars of different color), which is evaluated in terms of predicting co-complex membership (panels **a-c**), and correlation with functional distance (panels **d-f**) in the three networks that we study. Panels (a)-(c) display the number of co-complex pairs (as a fraction of the total number of co-complex pairs in the network) among pairs in the top percentile of each closeness ranking in the AC-Western (a), AC-MS (b), and Two-Hybrid (c) networks. Higher values indicate measures that are more biologically meaningful. Panels (d)-(f) display the average functional distance of pairs in the top percentile of each closeness ranking in the AC-Western (d), AC-MS (e), and Two-Hybrid (f) networks. Lower values indicate measures that are more biologically meaningful. Click here for file

Additional file 5 **Evaluating different ways of defining *PageRank Affinity *by using a weighted geometric mean to combine the two PageRank contributions**. We evaluate different ways of combining *c*_1 _= pr(*a *→ *b*) and *c*_2 _= pr(*b *→ *a*) using a weighted geometric mean: max(*c*_1_, *c*_2_)^*β*^·min(*c*_1_, *c*_2_)^1-*β*^. Each value of *β *gives a distinct closeness measure (represented by bars of different color), which is evaluated in terms of predicting co-complex membership (panels **a-c**), and correlation with functional distance (panels **d-f**) in the three networks that we study. Panels (a)-(c) display the number of co-complex pairs (as a fraction of the total number of co-complex pairs in the network) among pairs in the top percentile of each closeness ranking in the AC-Western (a), AC-MS (b), and Two-Hybrid (c) networks. Higher values indicates measures that are more biologically meaningful. Panels (d)-(f) display the average functional distance of pairs in the top percentile of each closeness ranking in the AC-Western (d), AC-MS (e), and Two-Hybrid (f) networks. Lower values indicate measures that are more biologically meaningful. Click here for file
