# Supplementary material for: Spectral affinity in protein networks
Source: BMC Syst Biol. 2009 Nov 29;3:112. doi: 10.1186/1752-0509-3-112 (PMC2797010; doi:10.1186/1752-0509-3-112)
Supplement: Additional file 1 — Predicted false negative interactions in the AC-Western network. The table lists disconnected node pairs with highest PageRank Affinity in the AC-Western network, along with evidence for the existence of each interaction. Each row specifies a pair of proteins and the rank of their PageRank Affinity score (closeness rank). For each pair we list evidence of the existence of this interaction in the evidence of interaction column by writing co-complexed if the two proteins are annotated as co-complexed in [40], and writing ac-western, ac-ms, two-hybrid, and other if the pair is listed as interacting in BioGRID using Affinity Capture-Western, Affinity Capture-MS, Two-Hybrid, or any other type of experiment. [file 1752-0509-3-112-S1.PDF]

Predicted false negative interactions in the AC-Western network

orf1	orf2	closeness rank	evidence of interaction
YER007W	YFL037W	183	other
YLR272C	YBL097W	203	ac-ms
YIL009C-A	YBL064C	211	-
YNL117W	YKR009C	238	-
YNL026W	YMR060C	242	other
YML121W	YGR163W	259	ac-ms, other
YBR077C	YML121W	260	other
YKR010C	YGR159C	292	-
YOR371C	YAL056W	308	other
YOR265W	YOR349W	314	other
YDR362C	YOR110W	340	co-complex, ac-ms
YKR036C	YLL001W	345	two-hybrid, other
YOR219C	YER145C	382	-
YIR017C	YPL038W	389	co-complex
YPL120W	YLR360W	394	ac-ms
YGR029W	YKR066C	398	-
YNL116W	YHR115C	401	ac-ms
YLL006W	YHR083W	408	-
YJR031C	YEL022W	422	other
YFL023W	YPR169W	431	two-hybrid
