# Supplementary material for: Spectral affinity in protein networks
Source: BMC Syst Biol. 2009 Nov 29;3:112. doi: 10.1186/1752-0509-3-112 (PMC2797010; doi:10.1186/1752-0509-3-112)
Supplement: Additional file 2 — Predicted false negative interactions in the AC-MS network. The table lists disconnected node pairs with highest PageRank Affinity in the AC-MS network, along with evidence for the existence of each interaction. Each row specifies a pair of proteins and the rank of their PageRank Affinity score (closeness rank). For each pair we list evidence of the existence of this interaction in the evidence of interaction column by writing co-complexed if the two proteins are annotated as co-complexed in [40], and writing ac-western, ac-ms, two-hybrid, and other if the pair is listed as interacting in BioGRID using Affinity Capture-Western, Affinity Capture-MS, Two-Hybrid, or any other type of experiment. [file 1752-0509-3-112-S2.PDF]

Predicted false negative interactions in the AC-MS network

orf1	orf2	closeness rank	evidence of interaction
YPL065W	YGR206W	62	-
YMR169C	YLR143W	66	-
YJR004C	YJR124C	77	-
YLR273C	YJL118W	89	-
YBR186W	YGL006W	103	-
YDR122W	YDR287W	109	-
YDR147W	YIL162W	111	-
YIL074C	YHR215W	154	-
YEL021W	YNL256W	156	-
YDL090C	YGL155W	160	-
YEL005C	YKL061W	187	-
YMR026C	YLR191W	237	ac-western, two-hybrid, other
YNR055C	YLL061W	305	-
YDR244W	YLR191W	321	ac-western, two-hybrid, other
YDR244W	YMR026C	322	ac-western, other
YOR265W	YJR091C	330	two-hybrid
YDR425W	YDL113C	341	-
YHR104W	YDR339C	346	-
YML043C	YJL025W	351	co-complex, ac-western
YKL100C	YLR143W	408	-
