# Supplementary material for: Spectral affinity in protein networks
Source: BMC Syst Biol. 2009 Nov 29;3:112. doi: 10.1186/1752-0509-3-112 (PMC2797010; doi:10.1186/1752-0509-3-112)
Supplement: Additional file 3 — Predicted false negative interactions in the Two-Hybrid network. The table lists disconnected node pairs with highest PageRank Affinity in the Two-Hybrid network, along with evidence for the existence of each interaction. Each row specifies a pair of proteins and the rank of their PageRank Affinity score (closeness rank). For each pair we list evidence of the existence of this interaction in the evidence of interaction column by writing co-complexed if the two proteins are annotated as co-complexed in [40], and writing ac-western, ac-ms, two-hybrid, and other if the pair is listed as interacting in BioGRID using Affinity Capture-Western, Affinity Capture-MS, Two-Hybrid, or any other type of experiment. [file 1752-0509-3-112-S3.PDF]

Predicted false negative interactions in the Two-Hybrid network

orf1	orf2	closeness rank	evidence of interaction
YOR086C	YML072C	156	other
YLR043C	YGR209C	185	other
YBR125C	YBL056W	197	-
YLR072W	YLR403W	228	-
YGL028C	YOR276W	234	-
YBR127C	YOR270C	238	co-complex, ac-western, ac-ms, other
YOR025W	YGR017W	243	-
YOL139C	YDR482C	246	-
YKL023W	YDR382W	265	-
YCR004C	YBR052C	293	ac-ms
YPR167C	YER042W	296	-
YLR287C	YLR340W	297	ac-ms
YDR044W	YOR330C	307	-
YEL065W	YOR330C	308	-
YEL065W	YDR044W	309	-
YMR261C	YML100W	310	ac-ms, other
YFL033C	YML100W	311	-
YFL033C	YMR261C	312	-
YNL183C	YMR178W	316	-
YER062C	YIL053W	317	other
