# Supplementary figures and images for: Spectral affinity in protein networks
Source: BMC Syst Biol. 2009 Nov 29;3:112. doi: 10.1186/1752-0509-3-112 (PMC2797010; doi:10.1186/1752-0509-3-112)

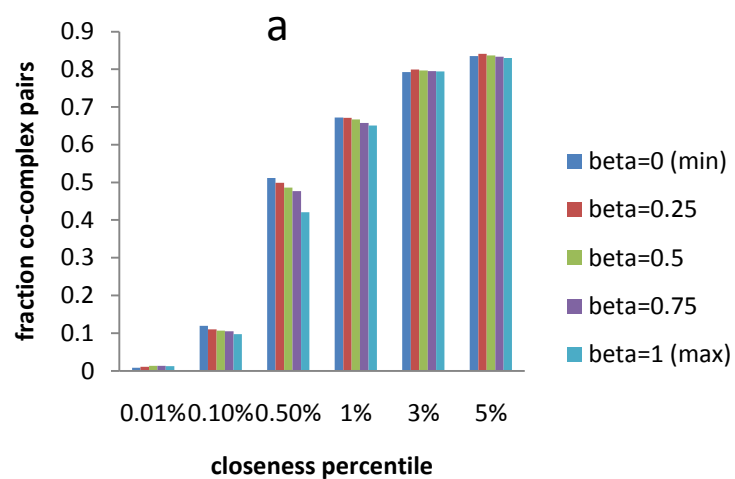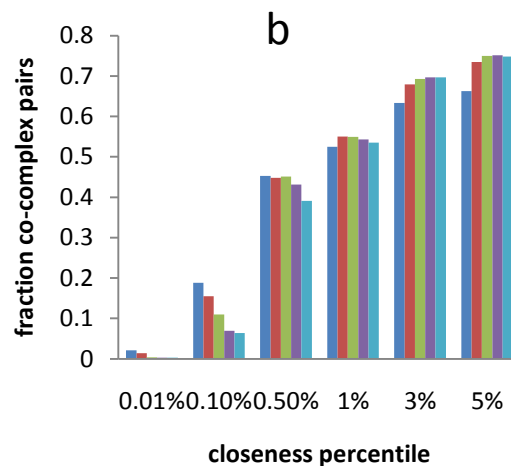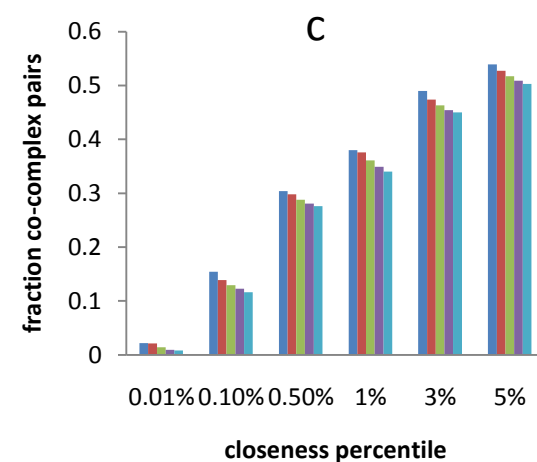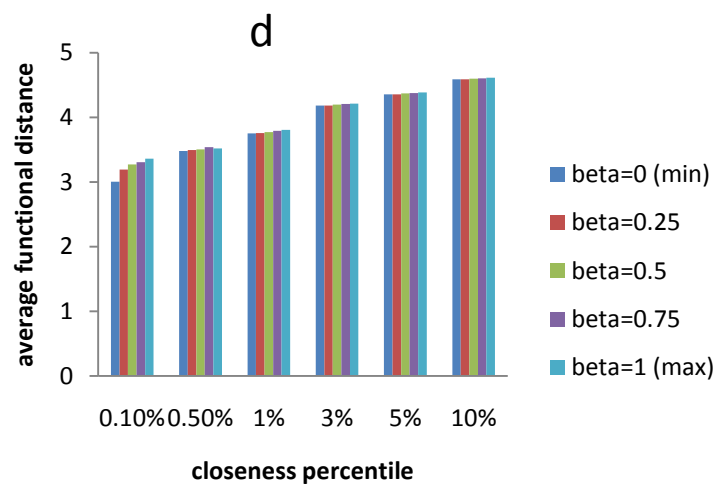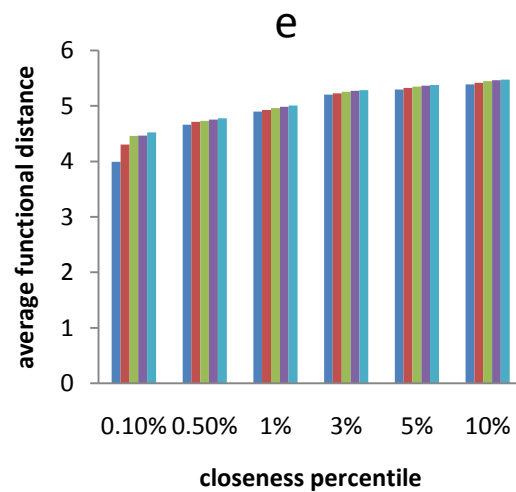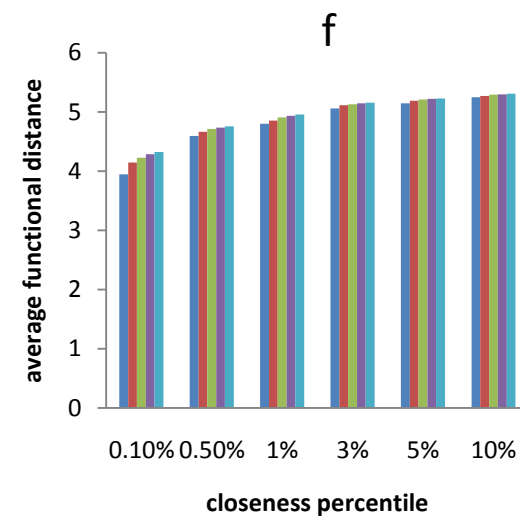

Supplement: Additional file 4 — Evaluating different ways of defining PageRank Affinity by using a weighted arithmetic mean to combine the two PageRank contributions. We evaluate different ways of combining c1 = pr(a → b) and c2 = pr(b → a) using a weighted arithmetic mean: β·max(c1, c2) + (1 - β)·min(c1, c2). Each value of β gives a distinct closeness measure (represented by bars of different color), which is evaluated in terms of predicting co-complex membership (panels a-c), and correlation with functional distance (panels d-f) in the three networks that we study. Panels (a)-(c) display the number of co-complex pairs (as a fraction of the total number of co-complex pairs in the network) among pairs in the top percentile of each closeness ranking in the AC-Western (a), AC-MS (b), and Two-Hybrid (c) networks. Higher values indicate measures that are more biologically meaningful. Panels (d)-(f) display the average functional distance of pairs in the top percentile of each closeness ranking in the AC-Western (d), AC-MS (e), and Two-Hybrid (f) networks. Lower values indicate measures that are more biologically meaningful. [file 1752-0509-3-112-S4.PDF]

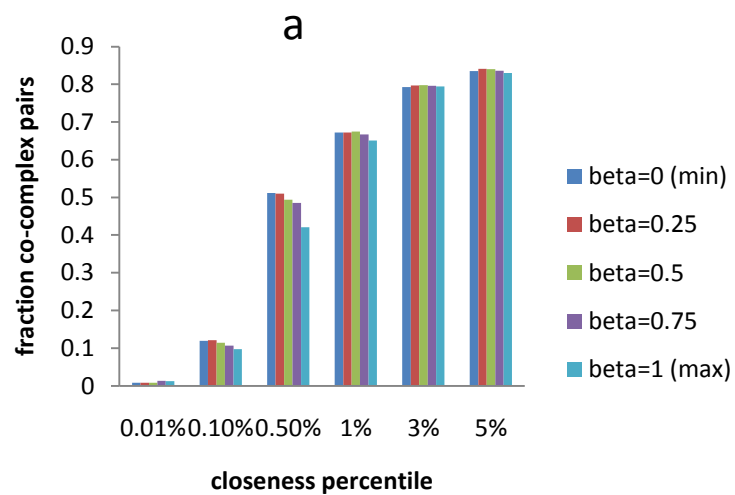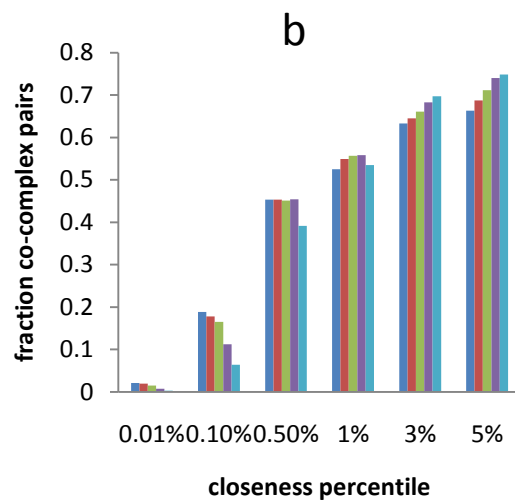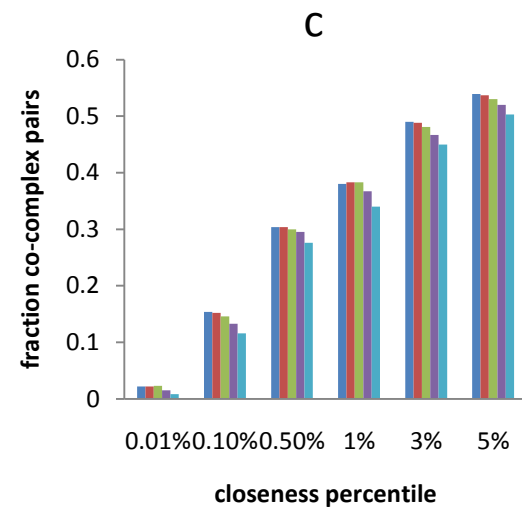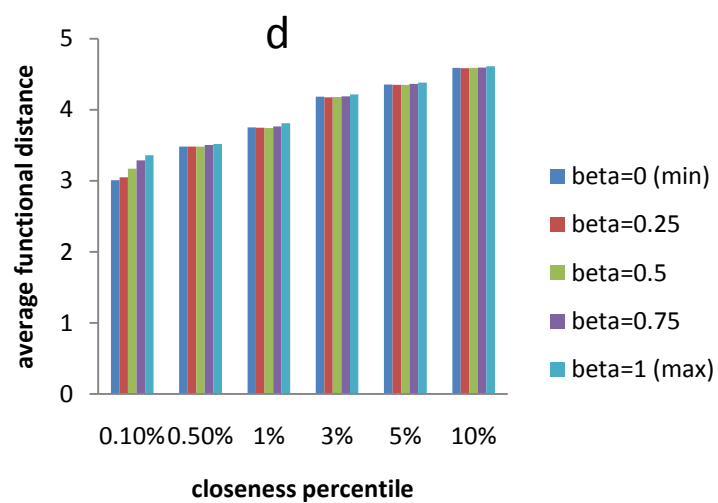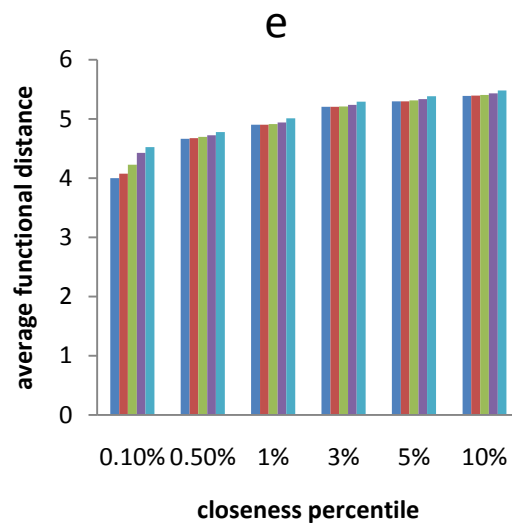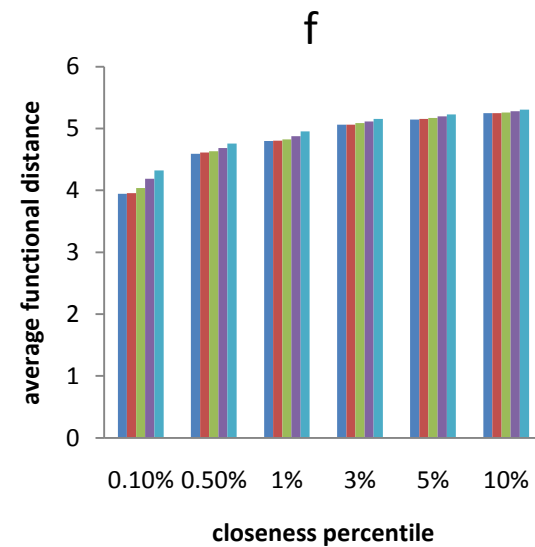

Supplement: Additional file 5 — Evaluating different ways of defining PageRank Affinity by using a weighted geometric mean to combine the two PageRank contributions. We evaluate different ways of combining c1 = pr(a → b) and c2 = pr(b → a) using a weighted geometric mean: max(c1, c2)β·min(c1, c2)1-β. Each value of β gives a distinct closeness measure (represented by bars of different color), which is evaluated in terms of predicting co-complex membership (panels a-c), and correlation with functional distance (panels d-f) in the three networks that we study. Panels (a)-(c) display the number of co-complex pairs (as a fraction of the total number of co-complex pairs in the network) among pairs in the top percentile of each closeness ranking in the AC-Western (a), AC-MS (b), and Two-Hybrid (c) networks. Higher values indicates measures that are more biologically meaningful. Panels (d)-(f) display the average functional distance of pairs in the top percentile of each closeness ranking in the AC-Western (d), AC-MS (e), and Two-Hybrid (f) networks. Lower values indicate measures that are more biologically meaningful. [file 1752-0509-3-112-S5.PDF]
